# Platinum deposition on functionalised graphene for corrosion resistant oxygen reduction electrodes†

**DOI:** 10.1039/d2ta03487e

**Published:** 2022-08-31

**Authors:** Noelia Rubio, Theo Suter, Zahra Rana, Adam J. Clancy, Seigo Masuda, Heather Au, Gabriel Coulter, Pichamon Sirisinudomkit, Paul F. McMillan, Christopher A. Howard, Cecilia Mattevi, Dan J. L. Brett, Milo S. P. Shaffer

**Affiliations:** Department of Organic and Inorganic Chemistry, University of Alcala Madrid 28802 Spain noelia.rubio@uah.es; Electrochemical Innovation Lab, Department of Chemical Engineering, University College London London WC1H 0AJ UK; Department of Chemistry, MSRH, Imperial College London W12 0BZ UK m.shaffer@ic.ac.uk; Department of Mining and Materials Engineering, Faculty of Engineering, Prince of Songkla University Hat Yai 90110 Songkhla Thailand; Department of Materials, Imperial College London SW7 2AZ UK; Department of Chemical Engineering, Imperial College London London SW7 2AZ UK; Department of Chemistry, University College London London WC1H 0AJ UK; Department of Physics and Astronomy, University College London London WC1H 0AJ UK

## Abstract

Graphene-related materials are promising supports for electrocatalysts due to their stability and high surface area. Their innate surface chemistries can be controlled and tuned *via* functionalisation to improve the stability of both the carbon support and the metal catalyst. Functionalised graphenes were prepared using either aryl diazonium functionalisation or non-destructive chemical reduction, to provide groups adapted for platinum deposition. XPS and TGA-MS measurements confirmed the presence of polyethyleneglycol and sulfur-containing functional groups, and provided consistent values for the extent of the reactions. The deposited platinum nanoparticles obtained were consistently around 2 nm *via* reductive chemistry and around 4 nm *via* the diazonium route. Although these graphene-supported electrocatalysts provided a lower electrochemical surface area (ECSA), functionalised samples showed enhanced specific activity compared to a commercial platinum/carbon black system. Accelerated stress testing (AST) showed improved durability for the functionalised graphenes compared to the non-functionalised materials, attributed to edge passivation and catalyst particle anchoring.

## Abstract 2

Functionalisation of graphene with functional groups affords promising supports for electrocatalysts due to their improved durability and enhanced specific activity compared to commercial systems.

## Graphical abstract

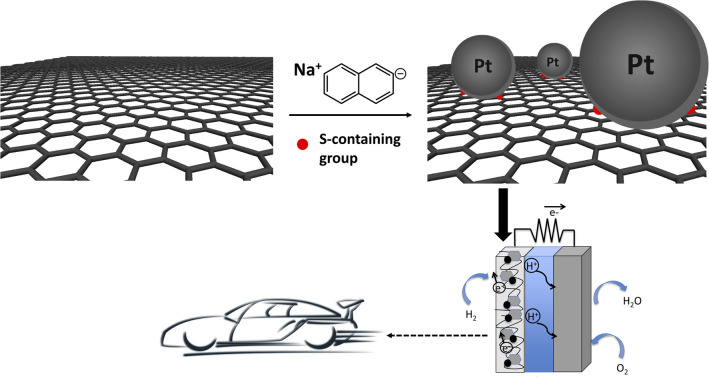

## 1 Introduction

There is an increased drive in the search for alternative energy sources to cope with society's growing demands. The development of polymer electrolyte fuel cells (PEMFCs) has the potential to resolve a number of global energy challenges.^1^ Current platinum-supported catalysts for the electrochemical oxygen reduction reaction (ORR) rely on costly carbon-supported platinum nanoparticles (generally, Pt/C catalysts with platinum loading up to 60 wt%) which undergo degradation over repeated cycling.^2^ The strong dependence on platinum-based catalysts for the challenging ORR reaction is one of the limitations on wider implementation of PEMFCs. Carbon black remains the most used support material to enhance Pt activity towards ORR; however, the support must supply conflicting demands, stabilising the hydrophilic platinum particles against ripening, whilst avoiding excessive water flooding, and suppressing oxidative degradation reactions. As an alternative to carbon black, graphene remains one of the most promising new materials in this field; its unique electrical properties make the electron transfer between graphene and substrates more favourable, whilst providing a very high accessible surface area.^3^ However, low Pt catalyst utilisation efficiency remains a challenge for carbon-based support materials. Different strategies have been studied to improve platinum utilisation, including the preparation of mesoporous platinum catalysts,^4^ reducing platinum loading^5^ or improving platinum dispersion.^6^ Functionalisation of the carbonaceous support can anchor the platinum particles to the surface, controlling both metal deposition and subsequent activity. Both covalent^7^ and non-covalent^8^ introduction of oxygen functionalities can provide sufficient interaction to increase the stability of the electrocatalyst on graphene. However, the nature of the functional group stabilising the Pt nanoparticle and the extent of functionalisation will affect the nanoparticle size and thus, the platinum efficiency.

This paper reports the synthesis of several graphenes functionalised with a variety of functional groups using a reductive chemistry approach^9–11^ and the traditional aryl diazonium functionalisation.^12^ The chosen functional groups contained moieties that can increase Pt nanoparticle stabilisation whilst improving graphene dispersibility in organic solvents. The impact of the type of functionality on the efficiency of the material as an ORR catalyst was subsequently assessed.

## 2 Experimental section

### 2.1 Materials

Few-layer graphene (FLG) feedstock was obtained from Cambridge Nanosystems UK, with a carbon purity >99.5% and an average lateral size of 1.2 μm. All the graphite starting materials were used without any further purification, though dried thoroughly as described below. Dimethylacetamide (DMAc, Sigma-Aldrich) was dried over 20 vol% 4 Å activated molecular sieves. Naphthalene (99%, Sigma-Aldrich) was dried under vacuum in the presence of phosphorus pentoxide before use. Sodium (99.95%, ingot), sodium borohydride, bromo-thioanisole, aniline-2-sulfonic acid and potassium hexachloroplatinate were purchased from Sigma-Aldrich and used as received.

### 2.2 Experimental procedures

#### 2.2.1 Preparation of sodium-naphthalide solution

A stock sodium-naphthalide solution was prepared to allow for simple, accurate addition of sodium to the corresponding graphite starting material. 23 mg (1 mmol) sodium and 128 mg (1 mmol) dried naphthalene were added to 10 mL degassed anhydrous DMAc in a N_2_-filled glove box, and stirred for 1 day until all sodium had dissolved, forming a dark-green solution. Brominated 5 kDa mPEG (mPEG-Br) was prepared *via* a method described in literature.^13^ The polymer and bromo-thioanisole were dried under vacuum over phosphorus pentoxide.

#### 2.2.2 Synthesis of exfoliated FLG

A Young's tube containing FLG starting material (15 mg, 1.25 mmol carbon) and a magnetic stirrer bar was heated at 400 °C for 1 h under vacuum, and then kept under vacuum for 16 h at room temperature, before placing in a glove box. 1.04 mL of sodium-naphthalide solution was added to the Young's tube and the concentration of graphite in DMAc adjusted to 0.1 M by addition of 11.46 mL of DMAc (C/Na = 12, [Na] = 0.008 M). The suspension was stirred at room temperature for 1 day under N_2_. To discharge the product, dry O_2_/N_2_ (20/80%, ∼1 L) was bubbled into the solution for 15 min, then stirred overnight under dry O_2_/N_2_ to discharge any remaining charges. The mixture was filtered through a 0.1 μm PTFE membrane and washed thoroughly with DMAc, ethanol and water to remove any residual naphthalene and sodium salts formed during the reaction. The product was obtained as a dark powder and was suspended in ethylene glycol for further Pt deposition.

#### 2.2.3 Synthesis of FLG-PhS-CH_3_ and FLG-PEG

A Young's tube containing FLG starting material (15 mg, 1.25 mmol carbon) and a magnetic stirrer bar was heated at 400 °C for 1 h under vacuum, and then kept under vacuum for 16 h at room temperature, before placing in a glove box. 1.04 mL of sodium-naphthalide solution was added to the Young's tube and the concentration of graphite in DMAc adjusted to 0.1 M by addition of 11.46 mL of DMAc (C/Na = 12, [Na] = 0.008 M). The suspension was stirred at room temperature for 1 day under N_2_. Bromo-thioanisole (20.8 mg, 0.104 mmol) or brominated polyethylene glycol (173.3 mg, 0.034 mmol) was added to the final hybrid dispersion and the solution was stirred for 12 h. To discharge the product, dry O_2_/N_2_ (20/80%, ∼1 L) was bubbled into the solution for 15 min, then stirred overnight under dry O_2_/N_2_ to discharge any remaining charges. The mixture was filtered through a 0.1 μm PTFE membrane and washed thoroughly with DMAc, ethanol and water to remove any residual naphthalene and sodium salts formed during the reaction. The product was obtained as a dark powder and was suspended in ethylene glycol for further Pt deposition.

#### 2.2.4 Synthesis of FLG-PhSO_3_H

Exfoliated FLG (15 mg) was suspended in DI water and sonicated for 10 minutes. Subsequently, 200 μL of isoamyl nitrite and aniline-2-sulfonic acid (150 mg, 1.42 mmol) were added to the reaction. The mixture was stirred at 80 °C for 10 h. Functionalised FLG was filtered through a 0.1 μm PTFE membrane and washed thoroughly with water and ethanol to remove any side products. The modified FLG was obtained as a dark powder and was suspended in ethylene glycol for further Pt deposition.

#### 2.2.5 Platinum nanoparticle deposition

The corresponding functionalised graphene (15 mg, 1.25 mmol) and chloroplatinic acid hydrate (12.3 mg, 0.03 mmol, estimated to achieve a 40 wt% Pt loading) were mixed with ethylene glycol (15 mL) and briefly bath-sonicated (15 min, 50 W) to give a metastable suspension. The mixture was stirred for one hour, sodium borohydride was added (10 mg, 0.25 mmol) and then heated at 80 °C for 2 hours. The mixture was filtered through a 0.1 μm PTFE membrane and washed thoroughly with acetone to remove any residual platinum precursor. The corresponding Pt/deposited graphene was redispersed in isopropanol:water to prepare the catalyst ink.

#### 2.2.6 Catalyst ink formulation

Catalyst inks were formulated from the synthesised materials Pt/FLG, Pt/FLG-PEG, Pt/FLG-PhSO_3_H and Pt/FLG-PhS, and from commercial Pt/C. All inks were prepared by sonicating the materials in a 50 : 50 isopropanol : water mixture, at a concentration of 1 mg mL^−1^. Working electrodes were then prepared by drop-casting the inks onto polished glassy carbon electrodes with a Pt loading of 0.35 μg cm^−2^.

#### 2.2.7 Rotating disc electrode (RDE) setup

ORR performance was evaluated using a three-electrode test system, using a Pt wire counter electrode and a Ag/AgCl reference electrode. Accelerated stress tests (AST) were performed on all samples by cycling the potential between 0.79 and 1.39 V *vs.* Ag/AgCl (3.5 M KCl) for 30 000 cycles at 100 mV s^−1^ in a nitrogen saturated solution. AST was paused, and cyclic voltammetry (CV) and linear sweep voltammetry (LSV) performed at 0, 2000, 5000, 10 000 and 30 000 cycles at a scan rate of 20 mV s^−1^, under N_2_ and O_2_ saturated solutions, respectively, before conditioning and resuming the AST protocol. LSVs were acquired at an electrode rotation speed of 1600 rpm and all tests were performed in a 0.1 M aqueous solution.

### 2.3 Equipment and characterisation

Thermogravimetric analysis coupled with mass spectrometry (TGA-MS) was performed using a Mettler Toledo TGA/DSC 1 instrument integrated with a Hiden HPR-20 QIC EGA mass spectrometer under nitrogen atmosphere. Samples were held at 100 °C for 30 min, then heated from 100 °C to 850 °C at 10 °C min^−1^ (N_2_ flow rate = 60 mL min^−1^). X-ray photoelectron spectroscopy (XPS) data were recorded using a K-alpha^+^ XPS spectrometer equipped with an MXR3 Al Kα monochromated X-ray source (*hν* = 1486.6 eV). X-ray gun power was set to 72 W (6 mA and 12 kV). Charge compensation was achieved with the FG03 flood gun using a combination of low energy electrons and the ion flood source. Survey scans were acquired using 200 eV pass energy, 1 eV step size and 100 ms (50 ms × 2 scans) dwell times. All high-resolution spectra were acquired using 20 eV pass energy, 0.1 eV step size and 1 s (50 ms × 20 scans) dwell times. Samples were prepared by pressing the sample onto carbon-based double-sided tape. Pressure during measurement acquisition was ≤1 × 10^−8^ mbar. Atomic compositions were calculated from averaged spectra obtained from at least 3 areas per sample. Raman spectra were collected on a Renishaw inVia micro-Raman (1000–3000 cm^−1^), using a 50 mW 532 nm laser at 10% laser power. Statistical Raman data were obtained from measurements carried out in Streamline mode of at least 500 areas per sample. Samples were prepared by drop casting dispersions on a glass slide or silicon wafer. For transmission electron microscopy (TEM) analyses, samples were prepared by drop-casting dilute graphene dispersions onto lacey carbon coated Cu TEM grids (Agar Scientific). Analyses were performed in HAADF/STEM mode using a FEI-Titan Themis microscope equipped with a probe corrector and working at 200 kV. TEM was carried out using a JEOL2100Plus TEM at 200 kV operating voltage. Ambient X-ray diffraction (XRD) data was recorded on a PANalytical X'Pert PRO diffractometer operating at 40 kV and 40 mA, with CuKα (*λ* = 1.542 Å) radiation, at a scan rate of 0.085° s^−1^, step size of 0.0334°, and 2*θ* varying between 5° and 60°. Dried powder samples (510 mg) were mounted onto a zero-background Si sample holder (PANalytical Ltd, UK) and levelled to the height of the top of the holder using a glass slide.

## 3 Results and discussion

Few-layer graphene (FLG) was exfoliated using reductive chemistry following a standard methodology for grafting short molecules and polymers.^14–16^ Charged graphene or “graphenide” was further functionalised by simple addition of 4-bromo-thioanisole or polyethylene glycol in one single step. Thioether moieties are well-known platinum chelators^17^ which could be used to complex and stabilise platinum nanoparticles; indeed, the presence of thioether groups has been demonstrated to support the size-controlled synthesis of Pt and Au nanoparticles.^18^ Polyethylene glycol chains may improve the interaction between catalyst support and ionomer. An additional approach was followed using a classic radical addition protocol with a diazonium salt,^12^ in which previously exfoliated FLGs *via* reductive chemistry were modified with sulfonic groups. Diazonium functionalisation was chosen in this case as sulfonic moieties are not compatible with reductive chemistry conditions. Sulfonation of carbon-supported catalysts is an efficient way to increase the triple-phase boundaries.^19,20^

**Scheme 1:**
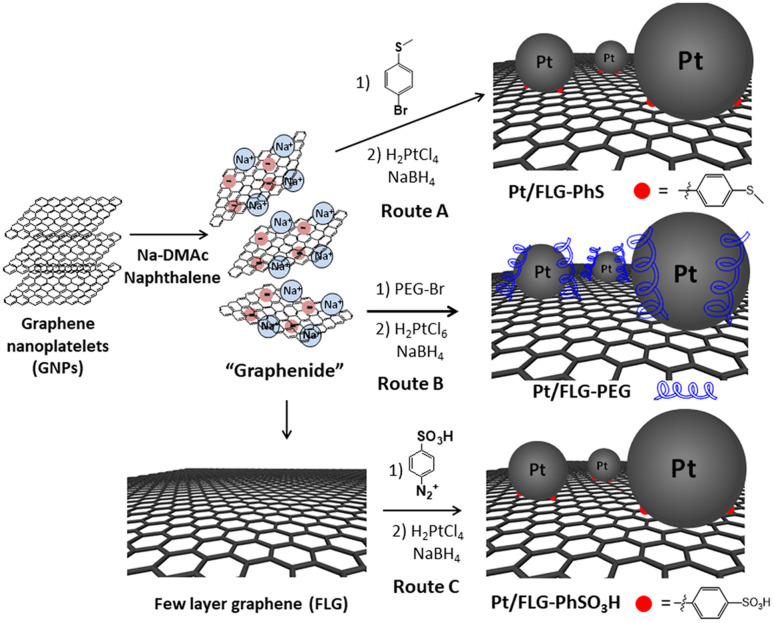
Different approaches to produce platinum supported-functionalised few layer graphenes.

The FLG starting material shows a low crystallinity and consists of small stacks of graphene (Fig. S1,† left panel), thus providing a highly porous material with a moderate number of edges, which can play a crucial role when using this material as catalyst support. The flake lateral size and number of layers are 1.2 ± 0.3 μm and 15, respectively. The exfoliation of ar-FLG was carried out using a standard methodology developed for grafting short alkyl groups on graphene layers.^11,14^ Sodium and naphthalene were used as the reducing agent and transfer reagent, respectively. FLG starting material was exfoliated using a C/sodium ratio of 12; this ratio was previously found to be an optimum for graphene exfoliation/functionalisation.^1a^ FLG was treated with sodium naphthalide in dimethylacetamide (DMAc); DMAc is a good solvent to exfoliate graphite as it can form ternary sodium graphite intercalation compounds (GIC).^21^ This reductive exfoliation route achieves a stable dispersion of exfoliated FLG as the charged layers are solvated in the polar solvent.^22^ The dark green colour of the sodium naphthalide disappeared after addition to the FLG due to electron transfer from the naphthalide to the graphite feedstock. The “graphenide” was either discharged with dry air for further characterisation or functionalised with 4-bromo-thioanisole (Scheme 1, route A) or with a bromine-terminated polyethylene glycol chain (Scheme 1, route B).

**Fig. 1:**
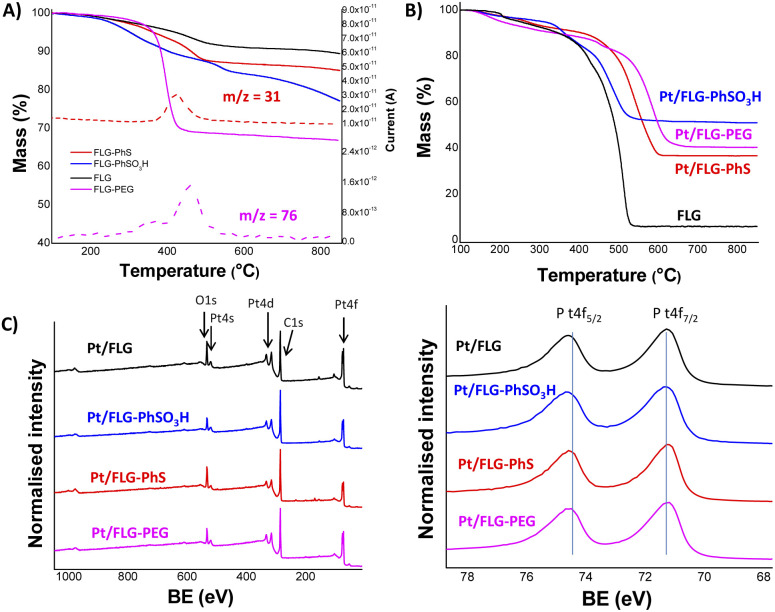
Characterisation of functionalised graphene samples. (A) TGA-MS under N_2_ of functionalised graphene samples. Phenyl and PEG fragments *m*/*z* 76 (C_6_H_4_^+^) and *m*/*z* 31 (CH_3_O^+^), respectively. (B) TGA under air of platinum supported graphene samples. (C) XPS spectra of platinum supported graphene samples (left panel) and platinum Pt 4f component (right panel).

The resulting products were characterised using TGA-MS under nitrogen and XPS. The FLG starting material shows a small mass loss (2.8 wt%) in the range from 100 °C to 800 °C (Fig. S2†). TGA-MS of FLG-PhS showed the expected fragment (*m*/*z* = 76) from the phenyl ring (Fig. 1A) accompanying the mass loss at around 450 °C. A grafting ratio (mass of additional groups relative to the carbonaceous framework) of 15 wt% correlated with a grafting density of one functional group every 91 carbon atoms (Table 1). Functionalisation with higher molecular weight polyethylene glycol groups correlated with the expected fragment (*m*/*z* = 31) and afforded a higher grafting ratio of 32 wt% but a lower grafting density of one functional group every 1287 carbon atoms, due to steric bulk, which correlates with previous results grafting polymers to graphenes.^23^ The FLG was functionalised with sulfonic groups following a radical addition reaction between previously exfoliated graphene and diazonium salt formed *in situ* from benzyl aniline sulfonic acid (Scheme 1, route C). Grafting ratios (19 wt%) were similar to the sample functionalised with thioether groups, corresponding to one functional group every 89 carbon atoms. Elemental analysis extracted from XPS measurements was consistent with the grafting ratios obtained from TGA-MS (Table 1 and Fig. 1C), as has been reported previously in other systems.^9^ X-ray diffraction analysis (CuK_α_ = 1.542 Å) of the starting material displayed a broad graphitic 002 layer peak (2*θ* = 26.6°), indicative of short stacks (Fig. S1, left panel†) consisting of 15 layers. This signal, corresponding to a layer spacing of 3.4 Å, became broader after reductive exfoliation and functionalisation (Fig. S1, right panel†), corresponding to a decrease in the number of layers per stack to 11–13 layers, demonstrating the extent of the exfoliation (Table 1).

**Table 1:** XPS quantification data of platinum deposited samples (weight percentage)

Sample	Number of layers^a^	%C	%O	%S	%Pt^b^ (wt)	%Pt^c^ (wt)	Grafting density^b^	Grafting density^c^	Pt NP size^d^ (nm)
ar-FLG	15				—	—	—	—	—
Commercial Pt/C	—	—	—	—	—	40	—	—	3.3 ± 0.34
Pt/FLG	12	83.4	9.5	—		52.1	—	—	2.64 ± 0.56
Pt/FLG-Ph-SO_3_H	13	79.2	14.6	1.9	42.1	45.7	89	68	3.8 ± 1.2
Pt/FLG-PhS	11	86.5	6.9	1.1	53.5	48.1	91	189	2.1 ± 0.28
Pt/FLG-PEG	12	83.9	9.7	—	51.4	52.0	1287	1460	2.02 ± 0.38

a Calculated from XRD.

b Calculated from TGA.

c Calculated from XPS.

d Values obtained from TEM measurements.

**Fig. 2:**
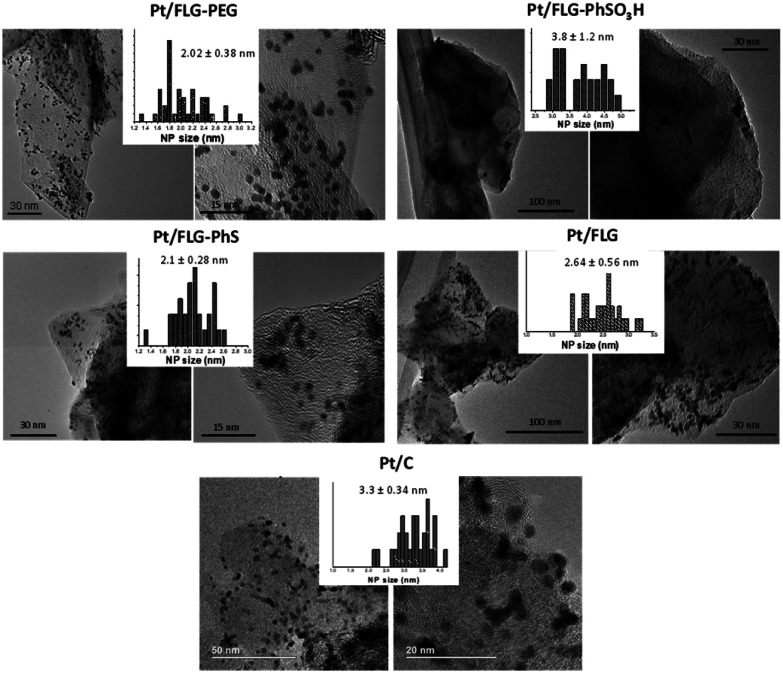
TEM images of the different functionalised few layer graphenes after platinum deposition. Histograms show the average Pt particle size and the standard deviation.

Platinum nanoparticles were deposited following the traditional route with chloroplatinic acid and ethylene glycol, aiming at 40 wt% loading relative to the FLG support.^24^ The amount of platinum was quantified using XPS and TGA under air atmosphere (Fig. 1B and C), with values obtained from both techniques in close agreement. In XPS, the platinum component (Fig. 1C, right panel) shows the typical Pt(0) 4f_5/2_ and 4f_7/2_ peaks (74.6 and 71.3 eV, respectively), confirming the presence of metallic platinum. Pt nanoparticle size was measured using TEM (Fig. 2) for comparison to commercial Pt/C catalysts. Pt/C commercial catalyst showed Pt nanoparticle values of about 3.3 ± 0.34 nm. However, products obtained from reductive chemistry functionalisation showed a smaller and consistent particle size (2 ± 0.4 nm and 2.1 ± 0.3 nm for PhS and PEG-functionalised graphenes, respectively), very similar to the optimum reported Pt nanoparticle size (2.2 nm) for ORR mass activities.^25^ On the other hand, the Ph-SO_3_H derivative obtained from aryl diazonium functionalisation showed a larger particle size (3.8 ± 1.2 nm). Pt nanoparticle aggregation in this case could be due to a less uniform distribution of functional groups for this reaction compared to reductive functionalisation;^26^ this heterogeneity might locate the particles only in highly functionalised areas of the graphene layer. Exfoliated FLG also showed a small particle size but slightly larger (2.6 ± 0.6 nm) than the reductively-functionalised samples, attributed to the absence of specific functional groups able to stabilise the Pt nanoparticles to the same extent, although the discharge process may introduce some oxygen groups in the absence of other reagents.^27^ The relationship between catalyst size and functional group of the FLG can be assigned to the widely reported influence of surface chemistries on Pt particle size during deposition.^28^

Further characterisation using X-ray diffraction measurements (CuK_α_ = 1.542 Å) confirmed the typical platinum face centered cubic (fcc) structures in all cases (Fig. S1,† right panel). ECSA is a measure of accessible Pt surface, which depends mainly on catalyst size and catalyst accessibility. Initial ECSA values measured *via* CV on the RDE (Table 2) showed that Pt/C possesses a significantly higher ECSA compared to the FLG materials despite having the second largest catalyst particle size. Pt/FLG-PEG and Pt/FLG-PhS had similar ECSA values and catalyst particle sizes, while Pt/FLG-PhSO_3_H showed lower ECSA and larger particle size. These three results for graphene based electrodes are internally consistent and can be explained simply by differences in Pt particle size. On the other hand, Pt/FLG has the lowest ECSA but not the largest catalyst particle size, suggesting that, in this case, Pt accessibility is the main limitation. The measured ECSA values of all FLG materials are significantly lower than the Pt/C commercial catalyst; this reduction can be assigned to the restacking of the graphitic sheets, reducing accessibility to the platinum particles, as observed in other graphene electrocatalyst systems.^29,30^ The extent of Pt utilisation follows the trend Pt/C > Pt/FLG-functionalised > Pt/FLG, suggesting that the functionalised groups play a useful role in preventing restacking of the graphene and the associated loss in initial ECSA.^31^

**Table 2:** Summary of ECSA values of the different functionalised FLG materials and commercial Pt/C before, during and after durability testing

ECSA m^2^ g(Pt)^−1^	Sample				
Cycles	Pt/FLG	Pt/FLG-PEG	Pt/FLG-PhSO_3_H	Pt/FLG-Ph-S	Pt/C
0	19.8	36.7	25.5	36.9	75.8
2000	14.7	22.4	22.3	27.6	59.9
5000	10.7	18.6	19.2	21.9	50.7
10 000	10.5	17.6	16.4	15	31.8
30 000	6.6	14.5	12	8.5	22.4

**Fig. 3:**
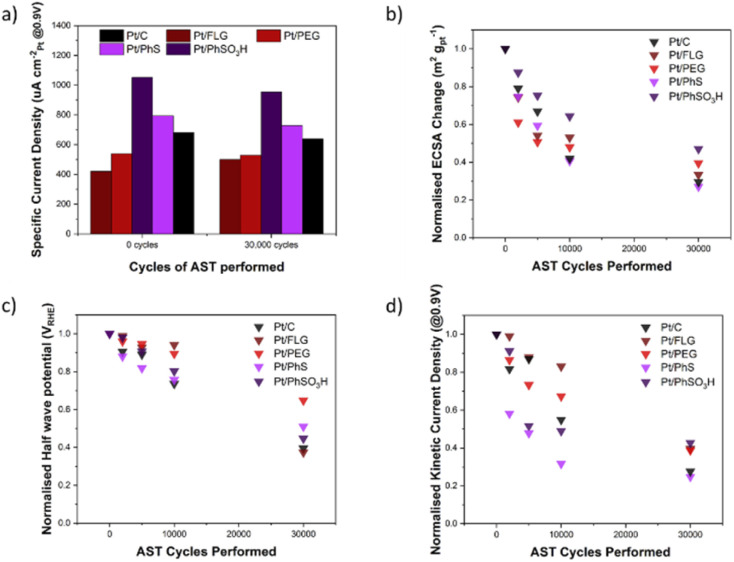
ORR parameters across the 1–1.6 V_RHE_ AST for different functionalised FLG materials and Pt/C over 30 000 corrosion cycles. (a) Specific current density (at 0.9 V) for each material at 0 and 30 000 cycles. (b) Normalised ECSA change across the 30 000 of AST. (c) Normalised half wave potential change across the 30 000 of AST. (d) Normalised kinetic current density (at 0.9 V) change across the 30 000 of AST.

ORR RDE experiments are typically compared *via* several different parameters across different parts of the LSV, these include *j*_k_ (current in kinetic controlled regime), *j*_d_ (current in diffusion-controlled regime) and half wave potential (potential at half *j*_d_); these parameters are reported in the ESI,† along with current density and Tafel plots for each material (Tables S1–S3 and Fig. S4, S5†). Fig. 3a shows the specific current density (μA cm^−2^_Pt_@0.9 V), a measure of current per available surface area of Pt. The initial specific current density of these electrocatalysts Pt/FLG-PhSO_3_H (1050 μA cm^−2^_Pt_) and Pt/FLG-PhS (790 μA cm^−2^_Pt_) outperformed commercial Pt/C (680 μA cm^−2^_Pt_), while Pt/FLG-PEG (540 μA cm^−2^_Pt_) and Pt/FLG (420 μA cm^−2^_Pt_) performed worse. It has been previously reported that functionalisation of support materials can lead to an enhancement of current density, due to the interaction between the functional group and the catalyst.^29,30^ The initial specific power density suggests that sulphur functional groups, particularly SO_3_H, improve catalytic performance.

Accelerated stress tests (AST) were performed between 1–1.6 V at 100 mV s^−1^ to target the stability of the carbon support. The AST was run between 0 and 30 000 but stopped at 0, 2 000, 5 000, 10 000 and 30 000 cycles to perform LSVs and CVs. The resulting specific current density, normalised change in *j*_k_, normalised change in half wave potential and normalised change in ECSA were determined (Fig. 3). The specific current densities of the different electrocatalysts did not change significantly from 0 to 30 000 cycles, indicating that the inherent catalyst activity and interaction between the functional groups/supports was not degraded.

The normalised ECSA change during AST (Fig. 3b) shows highest ECSA retention for the functionalised graphene materials Pt/FLG-PhSO_3_H and Pt/FLG-PEG, while Pt/FLG showed reduced catalyst surface area loss compared to commercial Pt/C. Loss of ECSA during the AST is usually attributed to two separate processes, carbon corrosion and catalyst ripening/agglomeration, that can be hard to distinguish. Particle agglomeration is widely reported to be significantly worse for low surface area carbons (LSAC) like highly graphitic support materials,^32^ due to the ease of agglomeration of Pt particles across the low surface area and limited microporosity of the supports. Graphene and FLG are examples of LSAC materials as they are entirely graphitic in nature and contain no inherent microporosity. As such, the agglomeration of platinum nanoparticles would be expected to cause more loss of ECSA on graphene materials compared to traditional support materials. Given the minor improvement in ECSA retention of the Pt/FLG over the Pt/C it is likely that the expected higher agglomeration in graphene samples is mitigated by improved support durability for the graphene samples. The improved ECSA durability in the functionalised samples (Pt/FLG-PhSO_3_H and Pt/FLG-PEG) can be attributed to anchoring of the platinum nanoparticles by the functional groups, thus reducing particle agglomeration, and secondly an improvement in carbon corrosion durability due to passivation of graphitic edges and holes.

Half-wave potential is commonly used as a benchmark indication of electrode film quality, and thus electrocatalytic system performance as it is dependent on both diffusion limiting and kinetic limiting process. Low half-wave potential may be an indication of diffusion limitations or electrode pore structure collapse, potentially a consequence of carbon corrosion of the support material. In this testing regime, half-wave potential is used to monitor overall electrode durability during the carbon corrosion targeted AST. The half wave potential retention (Fig. 3c) of the functionalised graphene materials (Pt/FLG-PhS and Pt/FLG-PhSO_3_H) was higher than the values obtained for commercial Pt/C, while Pt/FLG-PEG significantly outperformed all other samples.

Kinetic current density is highly dependent on the catalytic performance as it occurs soon after the overpotential and is limited almost entirely by kinetic concerns. Stability in this region is an indication of the functionalised support's capacity to limit kinetic resistances. The stability of kinetic current density (Fig. 3d) is much higher for Pt/FLG-PhSO_3_H, Pt/FLG and Pt/FLG-PEG samples, compared to commercial Pt/C and Pt/FLG-PhS. These results align closely to that for the normalised retention of ECSA (Fig. 3b), as access to catalyst surface area is one of the key limiting factors in the kinetic regime.

## 4 Conclusions

Modified graphenes with different functional groups were prepared either using a reductive chemistry approach or *via* aryl diazonium functionalisation. XPS and TGA-MS measurements confirmed the presence of the various functional groups, and provided consistent values for the extent of reaction.^9^ Platinum nanoparticles were successfully deposited using the traditional chloroplatinic acid/ethylene glycol route; TEM images showed a small and ideal particle size (around 2 nm) and uniform particle distribution across the graphene layers for the reductive functionalisation products. Pt particle size was larger for the sulfonated graphene obtained from radical addition (in the range of 4 nm). Reductive functionalisation achieved a more controlled functional group coverage, thus achieving a good particle stabilisation and ideal size compared to the product obtained from the radical addition reaction. Accelerated stress testing showed an improved durability for the functionalised graphenes compared to the non-functionalised FLG. The effect can be attributed not only to the stabilisation of edges and holes by the functional groups which makes these sites less reactive and more stable against corrosion (the case for Pt/FLG-PEG), but also to the inherent capacity of functional groups to anchor catalyst particles and reduce agglomeration (the case for Pt/FLG-PhSO_3_H and Pt/FLG-PEG). In general, ECSA values were significantly lower than for the commercial Pt/C catalyst due to graphene restacking, thus reducing Pt nanoparticle accessibility. The introduction of additives that improve layer separation could increase platinum utilisation whilst the functional groups help with support and catalyst durability. Reductive functionalisation is shown here as an attractive, versatile process to design and synthesise materials with specific functional groups. The choice of functional groups can provide targeted properties not only as catalyst supports as shown here, but for any application where specific surface interactions are beneficial, such as when designing platforms for sensing^33^ or biomedical technologies.^34^

## Conflicts of interest

There are no conflicts to declare.

## Supplementary Material

TA-010-D2TA03487E-s001
