# Supplementary material for: Platinum deposition on functionalised graphene for corrosion resistant oxygen reduction electrodes
Source: J Mater Chem A Mater. 2022 Aug 31;10(37):20121–7. doi: 10.1039/d2ta03487e (PMC9514556; doi:10.1039/d2ta03487e)
Supplement: TA-010-D2TA03487E-s001 [file TA-010-D2TA03487E-s001.pdf]

Electronic Supplementary Material (ESI) for Journal of Materials Chemistry A.
This journal is © The Royal Society of Chemistry 2022

Platinum deposition on functionalised graphene for corrosion resistant oxygen reduction electrodes

Noelia Rubio,[a,c*‡] Theo Suter,[b ‡] Zahra Rana,[b] Adam J. Clancy,[f,g] Seigo Masuda,[e] Heather Au,[f] Gabriel Coulter,[c] Pichamon Sirisinudomkit,[c,d] Paul F. McMillan,[g] Christopher A. Howard,[h] Cecilia Mattevi,[e] Dan J.L. Brett[b] and Milo S.P. Shaffer[c,e*]

a. *Department of Organic and Inorganic Chemistry, University of Alcala, Madrid, 28802, Spain.*

b. *Electrochemical Innovation Lab, Department of Chemical Engineering, University College London, London, WC1H 0AJ, U.K.*

c. *Department of Chemistry, MSRH, Imperial College London, W12 7SL U.K.*

d. *Prince of Songkla university, Thailand*

e. *Department of Materials, Imperial College London, SW7 2AZ, U.K.*

f. *Department of Chemical Engineering, Imperial College London, London, SW7 2AZ, U.K.*

g. *Department of Chemistry, University College London, London, WC1H 0AJ, U.K.*

h. *Department of Physics and Astronomy, University College London, London, WC1H 0AJ, U.K.*

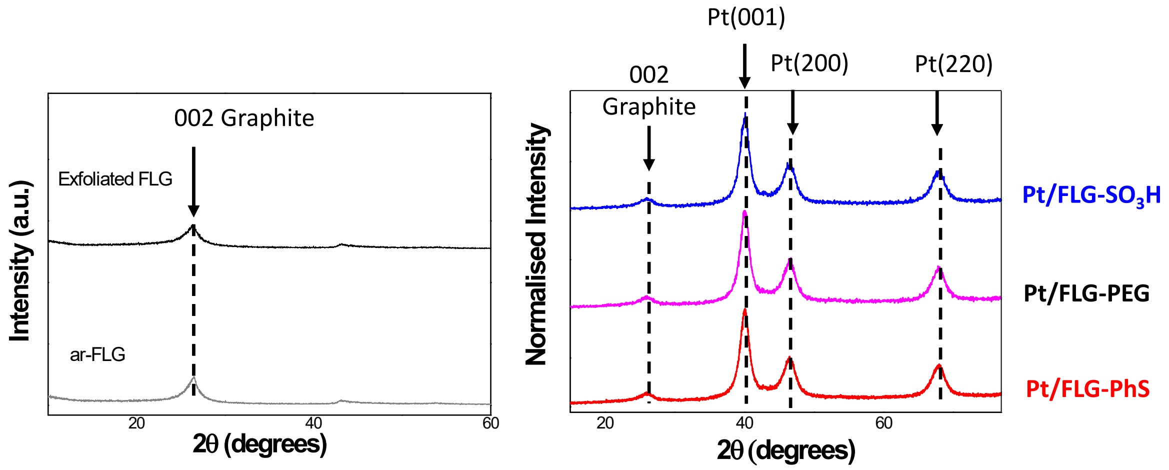

Figure S1. XRD patterns of as-received and exfoliated FLG (left panel) and the different modified graphenes (right panel).

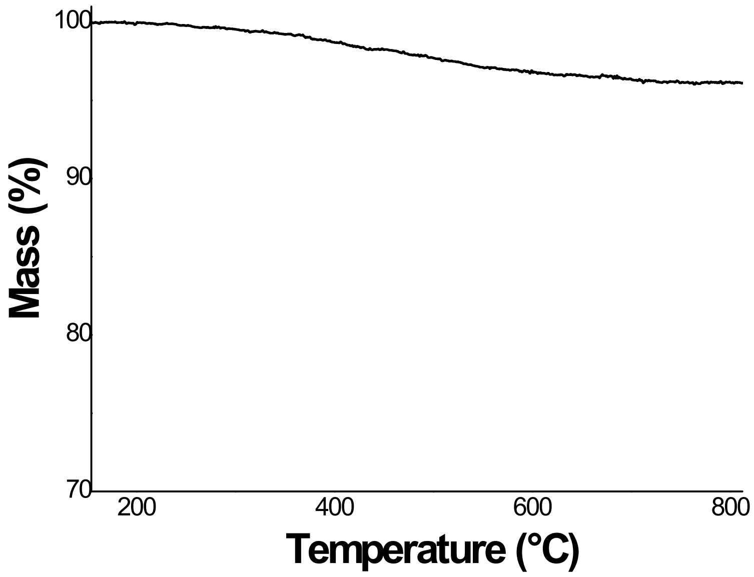

Figure S2. Thermogram under N_2 showing the degradation profile of as-received FLG material.

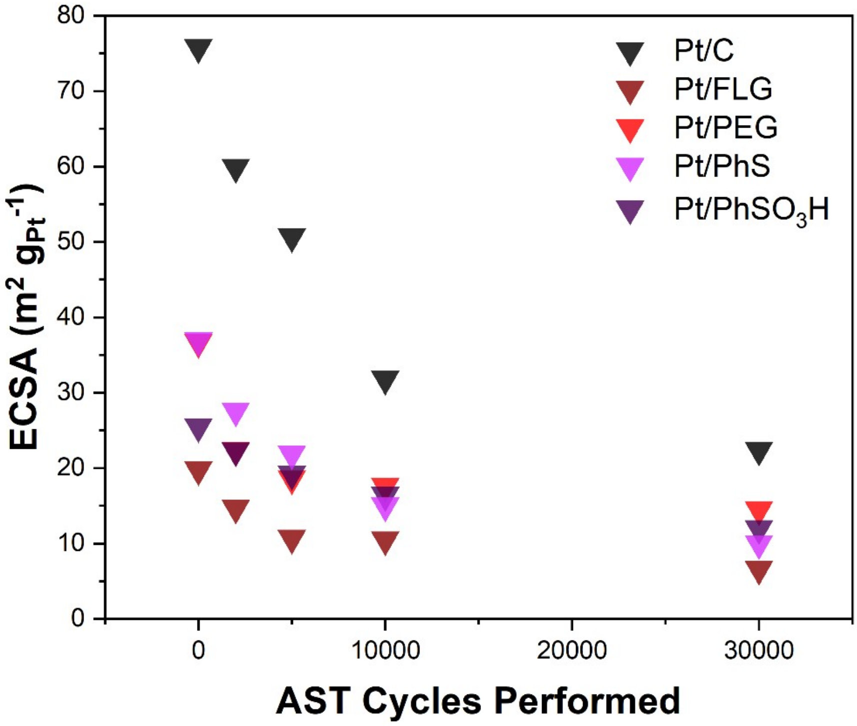

Figure S3. Graph comparing ECSA value change during durability testing of the different functionalised graphenes.

Table S1. Half wave potential voltage for each material during the AST experiments.

Half wave potential (V)	Sample				
Cycles	Pt/FLG	Pt/FLG-PEG	Pt/FLG-$PhSO_3H$	Pt/FLG-Ph-S	Pt/C
0	0.613	0.705	0.712	0.725	0.731
2000	0.606	0.677	0.627	0.711	0.662
5000	0.569	0.667	0.583	0.658	0.651
10,000	0.557	0.631	0.539	0.582	0.538
30,000	0.229	0.457	0.363	0.324	0.289

Table S2. Diffusion current at 0.4 V for each material during the AST experiments.

Diffusion current at 0.4V (mA cm^{-2})	Sample				
Cycles	Pt/FLG	Pt/FLG-PEG	Pt/FLG-$PhSO_3H$	Pt/FLG-Ph-S	Pt/C
0	5.40	5.55	5.60	5.48	6.63
2000	5.08	5.38	5.34	5.25	6.43
5000	4.9	5.44	5.60	4.85	6.29
10,000	4.88	5.15	5.4	4.58	5.47
30,000	3.85	4.73	5.34	4.16	5.34

Table S3. Kinetic current at 0.9V for each material during the AST experiments.

Kinetic current at 0.9V (mA cm^{-2})	Sample				
Cycles	Pt/FLG	Pt/FLG-PEG	Pt/FLG-$PhSO_3H$	Pt/FLG-Ph-S	Pt/C
0	0.02916	0.06925	0.09395	0.10259	0.18101
2000	0.02886	0.05986	0.08565	0.05967	0.81723
5000	0.02563	0.05077	0.04852	0.04909	0.15755
10,000	0.02422	0.04662	0.04599	0.03253	0.09937
30,000	0.01153	0.02685	0.0401	0.02545	0.05007

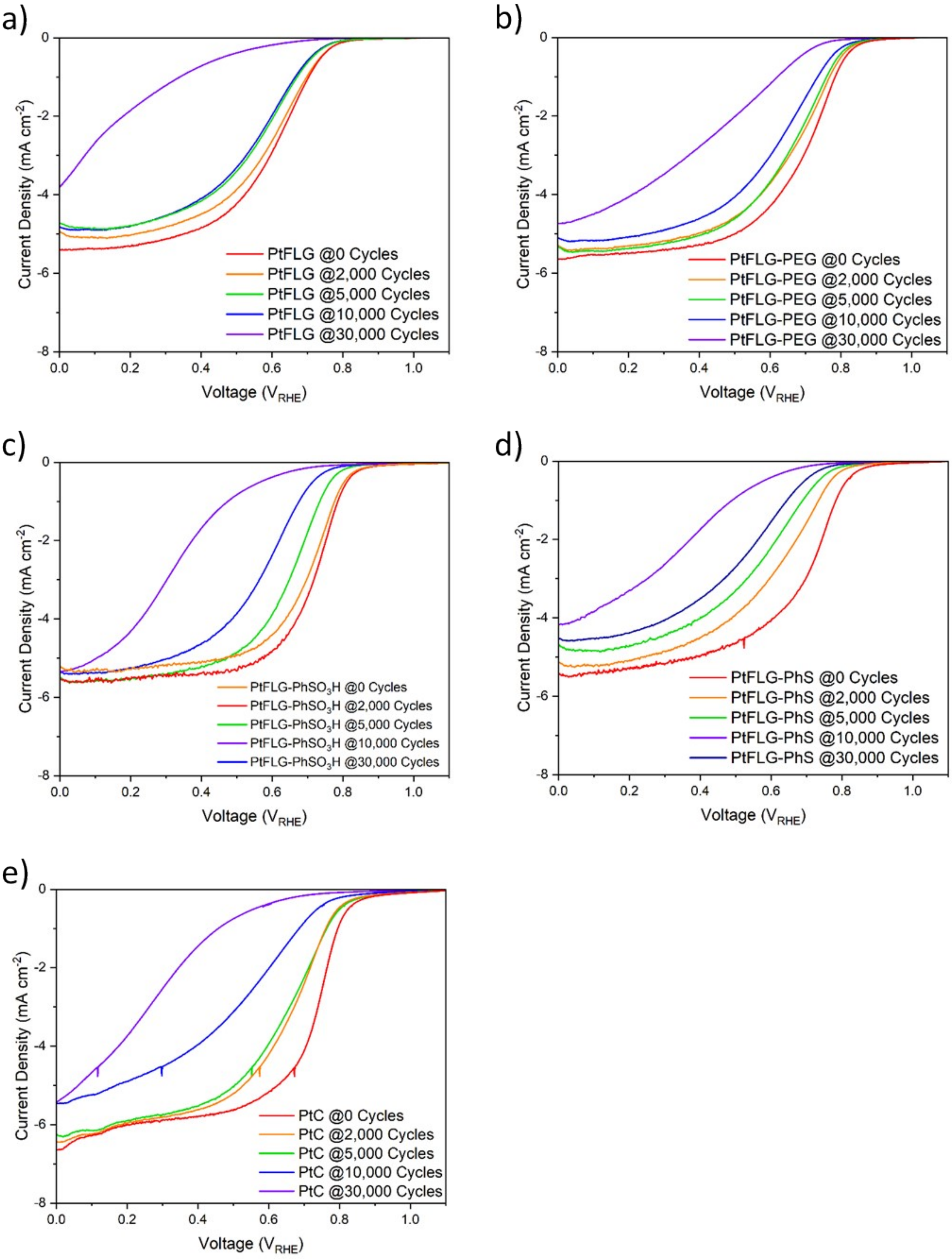

Figure S4. LSVs for every material at each stage of the AST

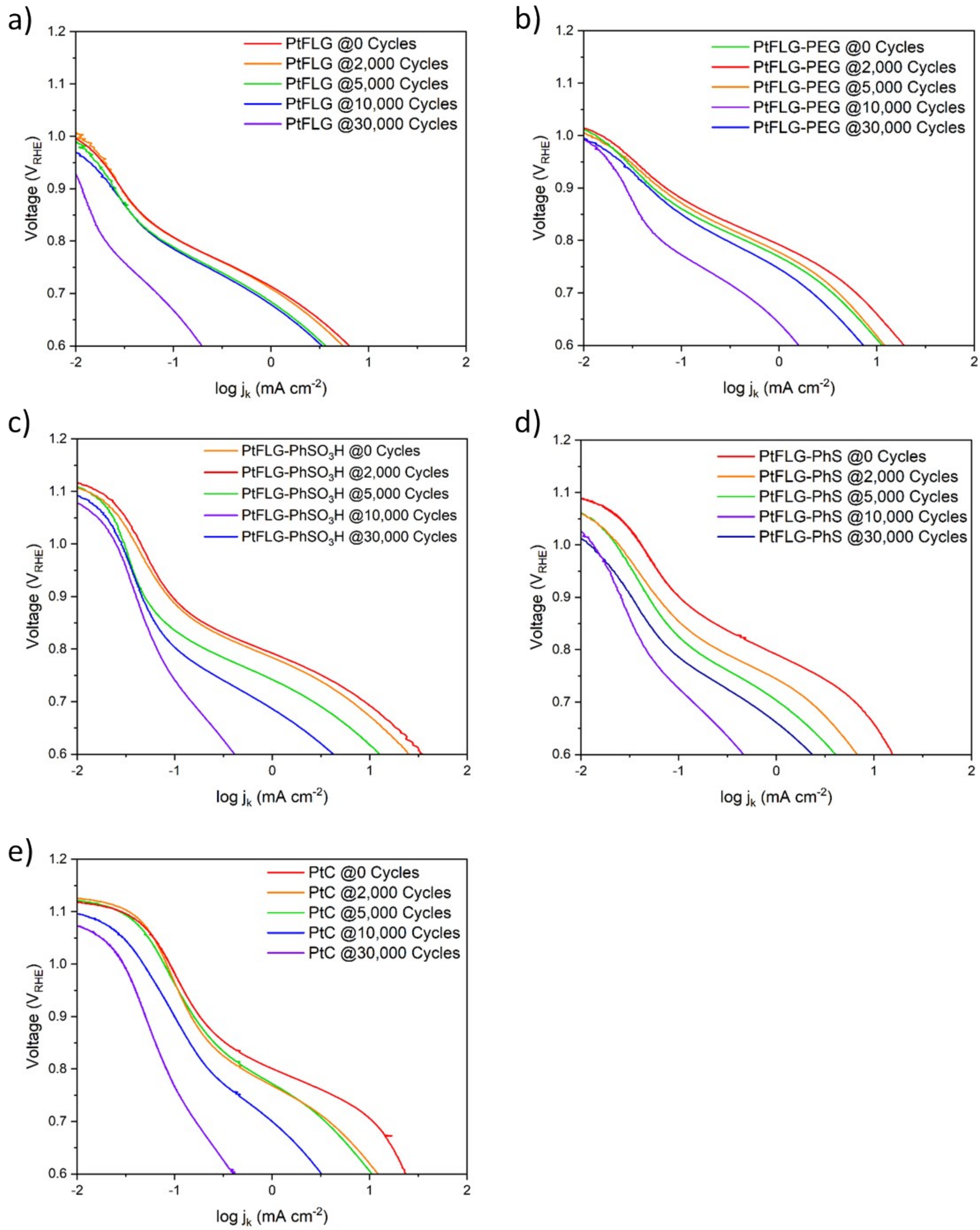

Figure S5. Tafel plots for every material at each stage of the AST
